# Pindborg tumor associated with a supernumerary tooth: a case report

**DOI:** 10.4322/acr.2021.358

**Published:** 2022-02-17

**Authors:** Hannah Gil de Farias Morais, Weslay Rodrigues da Silva, Ana Cláudia de Macêdo Andrade, Nelmara Sousa e Silva, Mariana Carvalho Xerez, José Wittor de Macêdo Santos, Adriano Rocha Germano, Antônio de Lisboa Lopes Costa

**Affiliations:** 1 Universidade Federal do Rio Grande do Norte, Department of Dentistry, Natal, RN, Brasil

**Keywords:** Odontogenic Tumors, Dental Tissue Neoplasms, Calcifying Epithelial Odontogenic Tumor, Pindborg tumor

## Abstract

The calcifying epithelial odontogenic tumor is a rare benign neoplasm that accounts for approximately 1% of all odontogenic tumors. Most of the cases occur in the posterior mandible, and a few involve the maxilla. Despite their relatively indolent biological behavior, tumors in the maxilla tend to grow fast. We report the case of a 33-year-old female patient exhibiting swelling in the right maxilla. An isodense area associated with an impacted supernumerary tooth was found on imaging examination. The histopathologic diagnosis was a calcifying epithelial odontogenic tumor. The treatment of choice was surgical removal of the lesion and associated dental elements. The patient has been followed up for 11 months and shows no signs of recurrence. Besides describing this case, we reviewed the literature on the association of calcifying epithelial odontogenic tumors with supernumerary teeth and found two case reports addressing this subject.

## INTRODUCTION

Odontogenic tumors are heterogeneous entities derived from tissues that constitute the tooth-forming apparatus, which may be of benign or malignant origin. According to the last classification of WHO (2017), these tumors are categorized based on their biological behavior and whether they derive from the epithelium or mesenchyme. The calcifying epithelial odontogenic tumor (CEOT) is classified as a benign neoplasm of epithelial origin, accounting for only 0.6% to 1.7% of all odontogenic tumors.^1^^,^ 2 This tumor was described for the first time in 1955 by the Danish pathologist Jens J. Pindborg and is therefore also known as Pindborg tumor.^2^

The etiopathogenesis of CEOT is still controversial, but the tumor is believed to arise from dental lamina remnants, from the reduced epithelium or intermediate layer of the enamel organ, or even from the oral epithelium.^3^^,^ 4 These tumors affect a wide age range but are more common between the fourth and sixth decades of life. No sex predilection has been reported. CEOTs are mostly intraosseous (96%), and the mandible is more affected than the maxilla (2:1).^5^

The diagnosis of COET is made by histopathological analysis of a fragment obtained preferentially through an incisional biopsy. Microscopically, the tumor appears as a benign neoplastic process of odontogenic epithelial origin characterized by the proliferation of polyhedral cells in an arrangement of sheets, islands, or nests amidst a stroma of fibrous connective tissue. Typically, the tumor contains amyloid-like material and basophilic concentric calcifications (Liesegang rings).^6^

Reports of cases of COET are scarce in the literature. The study of these rare tumors is important since it provides information that assists in the diagnosis, thus allowing better treatment planning and minimizing the risk of recurrence. This study aims to report an uncommon case of CEOT in the posterior maxilla associated with a supernumerary tooth.

## CASE REPORT

A 33-year-old female patient sought a referral center for oral-maxillofacial surgery, reporting a 15-year history of an asymptomatic swelling in the right maxilla, which started to exhibit ulceration about 8 months earlier. An extraoral physical examination revealed no alterations.

**Figure 1:**
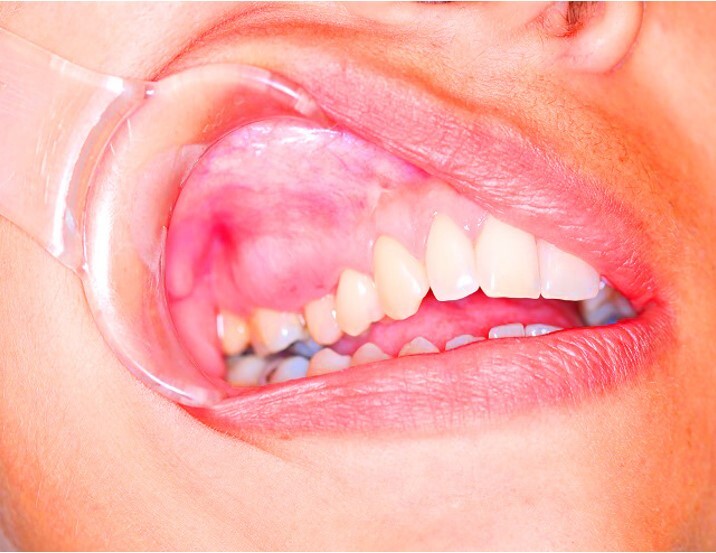
Clinical appearance of the lesion. Expansive mass in the right maxilla covered with normal-appearing oral mucosa.

On intraoral physical examination, a normochromic, hardened, sessile mass of exophytic growth was observed in the right maxillary region, with bulging along the bottom of the vestibule. The lesion was associated with ulcerations and paresthesia (Figure 1).

**Figure 2:**
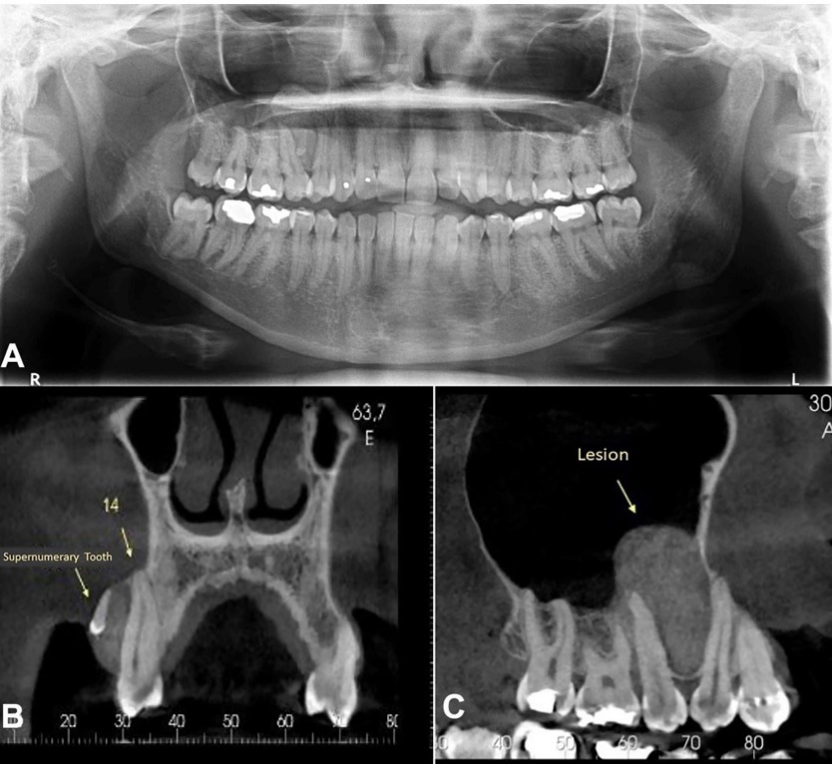
Imaging features of the lesion. **A** – Panoramic radiograph showing a unilocular lesion between the roots of right upper premolars and the presence of denticle-like mineralized material; **B** – Coronal view of cone-beam computed tomography (CBCT) image. Isodense lesion associated with upper right first premolar. Note the presence of a micro-tooth inside the tumor; **C** – Sagittal view of CBCT image. Isodense oval lesion in the right maxilla extending into the maxillary sinus and causing displacement of the roots of right upper premolars.

Panoramic radiography revealed a unilocular lesion surrounding a mineralized structure that resembled a denticle between the roots of the first and second right upper premolars (Figure 2A). Cone-beam computed tomography (CBCT) revealed expansive lesion growth into the maxillary sinus that caused the displacement of first and second right upper premolar extending to the upper right first molar. In addition, an impacted supernumerary tooth associated with the lesion was observed (Figure 2B-C). The diagnostic hypotheses were CEOT and central ossifying fibroma, and the patient was thus submitted to an incisional biopsy.

**Figure 3:**
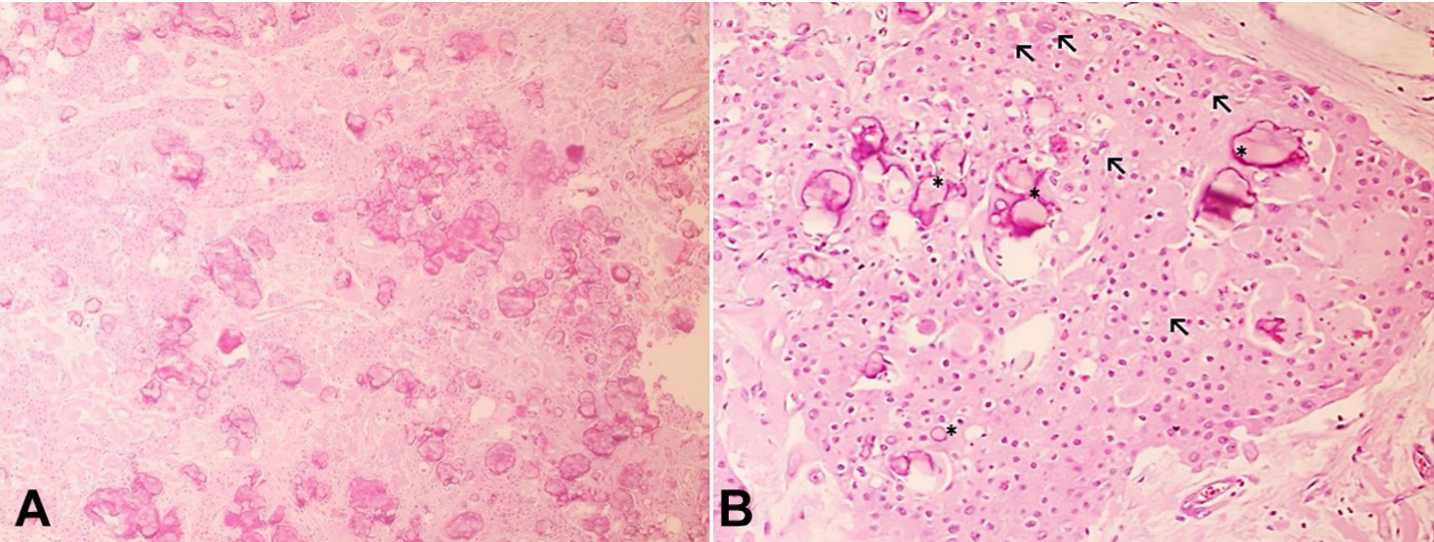
Histopathological features of the lesion. **A** – Sheets, islands and nests of neoplastic cells intermingled with irregular calcification foci (H&E, 100X); **B** – Polyhedral epithelial cells with eosinophilic cytoplasm exhibiting discrete cellular and nuclear pleomorphism (arrows), intercalated with homogenous eosinophilic material and concentric basophilic calcifications (asterisk) (H&E, 400X).

Histopathological analysis of the specimen showed the presence of sheets, islands, and sometimes nests of polyhedral odontogenic epithelial cells with well-defined contours amidst a stroma of fibrous connective tissue. The cells exhibited cellular and nuclear pleomorphism. The tumor also contained areas of amyloid-like eosinophilic amorphous material, as well as small irregular foci of concentric calcifications (Liesegang rings) (Figure 3A-B).

**Figure 4:**
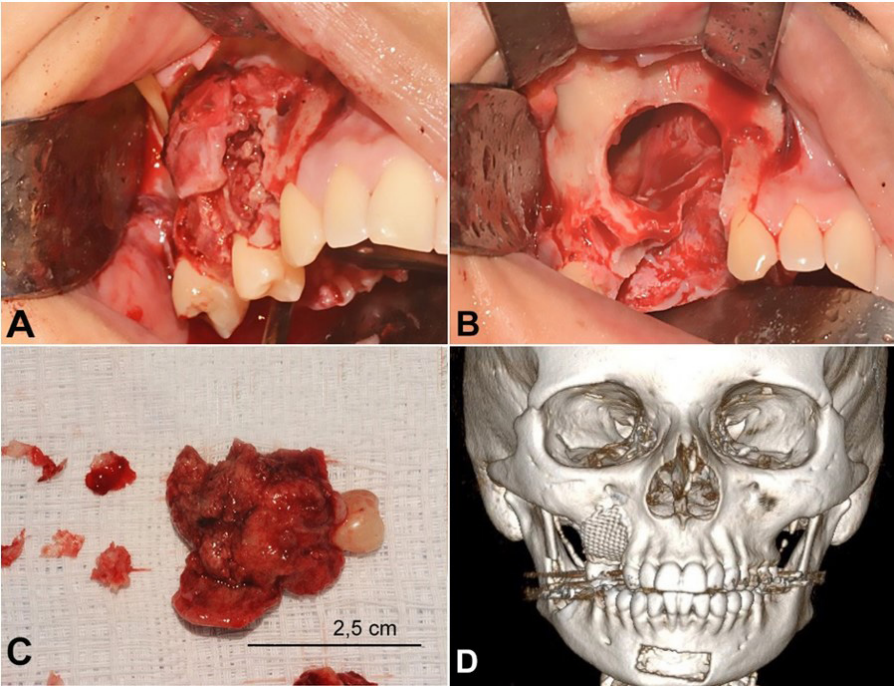
Intraoperative images and postoperative CT. **A** – Intraoperative view of the tumor involving buccal cortical bone in the region of upper right premolars and first molar; **B** – Intraoperative view of the bone cavity after curettage and removal of the involved teeth; **C** – Fragments of the surgical specimen sent for anatomopathological analysis; **D** – 3D-CT reconstruction image after repair of the maxillary defect.

The treatment of choice was enucleation followed by curettage and extraction of right upper premolars and first molar associated with the lesion. The maxillary defect was reconstructed using titanium meshes and autogenous corticomedullary bone grafts removed from the mandibular symphysis and right ramus (Figure 4A-D). A surgical specimen was sent for histopathological analysis, in which the microscopic findings confirmed the diagnosis of CEOT.

**Figure 5:**
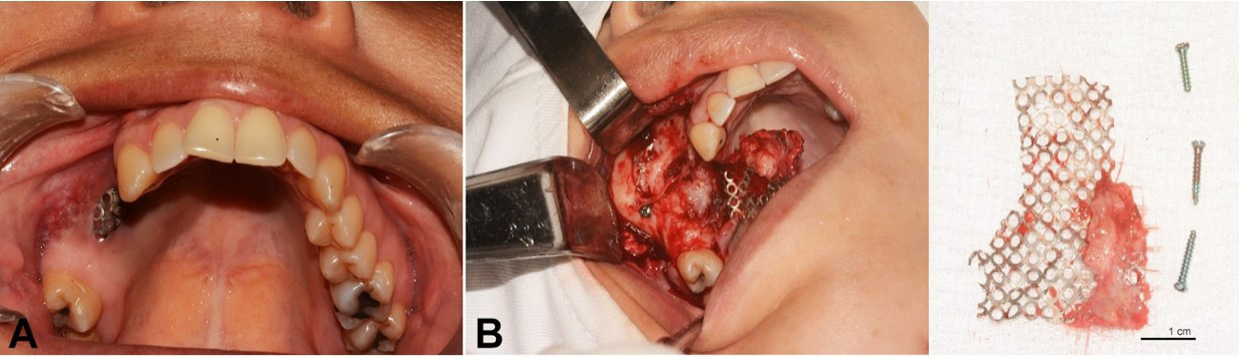
Intraoperative images after twelve months of the intraoral exposure of superficial titanium mesh. **A** – Intraoral view of superficial titanium mesh exposure. **B** – Intraoperative view of the superficial titanium mesh removal and titanium mesh and screws removed.

**Figure 6:**
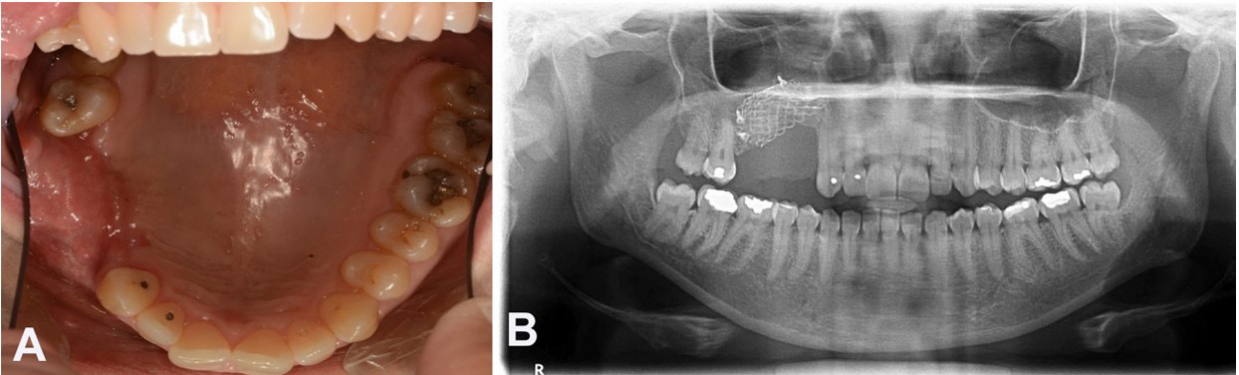
Follow-up images after thirteen months of the lesion removal. **A** – Intraoral view; **B** – Panoramic radiograph showing upper titanium mesh in position and no recurrence signs.

After 12 months postoperatively, the superficial titanium mesh was removed due to its intraoral exposure (Figure 5A-B). The upper mesh remained in position due to the graft's osseointegration, which acts as a physical barrier between the graft and the maxillary sinus. The patient has been under follow-up for 13 months and has shown no signs of recurrence (Figure 6A-B).

## DISCUSSION

In 2017, the World Health Organization (WHO) classified CEOT as a benign epithelial odontogenic tumor.^6^ Odontogenic tumors account for 2.2% to 3.3% of all lesions diagnosed at oral pathology services.^7^^,^ 8 CEOT corresponds to about 1% of these tumors and is, therefore, an uncommon neoplasm.^9^

Approximately 90% of CEOT cases are intraosseous, and the mandible is the most affected site in these patients. Maxillary tumors are observed in 25% to 41% of cases and generally occur in the posterior region.^10^^-^ 12 Peripheral CEOT accounts for about 10% of cases and has a predilection for the anterior gingiva.^10^^,^ 11 The present report adds to the small number of CEOT cases located in the maxilla since most cases occur in the posterior mandible.

There is no sex predilection for CEOT, and the tumor usually causes a painless, slow-growing swelling.^9^^,^ 10 ^,^ 13 ^,^ 14 However, tumors in the maxilla tend to grow rapidly and are not circumscribed.^15^ In the present case, the tumor exhibited a slow evolution and no symptoms; however, the exacerbation of growth in the last 8 months resulted in ulcerations and paresthesia. In approximately 50% of cases, CEOT is associated with an unerupted tooth.^14^^,^ 16 However, association with a supernumerary tooth as in the present case is rarely observed (Table 1).^12^^,^ 17

**Table 1:** Case reports of calcifying epithelial odontogenic tumor associated with supernumerary teeth

Ref	Sex/Age(y)	Location	Symptoms	Radiography	Evolution	Treatment	Follow-up
11	M/13	Anterior Maxilla	Absent	Mixed	ND	Enucleation	One year
16	F/45	Anterior Maxilla	Swelling	Radiolucent	Two months	Enucleation + Curettage	ND
Index case	F/33	Posterior Maxilla	Swelling/Paresthesia	Radiopaque	Fifteen years	Enucleation + Curettage	Eleven months

The clinical characteristics of CEOT are nonspecific. The radiographic features of CEOT vary according to the evolution time of the tumor, usually appearing as a radiolucent, uni- or multilocular lesion.^18^ Mineral deposition may occur, forming the characteristic radiopaque flakes.^3^^,^ 19 On imaging tests, the differential diagnosis of CEOT includes odontoma during maturation, central ossifying fibroma, and ameloblastoma.^3^^,^ 10 ^,^ 19 In the present case, the imaging findings led to the initial diagnostic hypotheses of CEOT and ossifying fibroma, in agreement with the literature.

The typical microscopic features of CEOT were identified in the present case: a proliferation of polyhedral cells of epithelial odontogenic origin arranged in sheets, islands, or nests amidst a stroma of fibrous connective tissue; extensive deposition of amyloid-like material; deposition of mineral material forming oval and concentric structures (Liesegang rings).^9^^,^ 11 ^,^ 19 Although the presence of clear cells in the parenchyma has been reported for some CEOTs, particularly those located in the maxilla, these cells were not observed in our case.^20^

CEOT can infiltrate bone trabeculae even in the radiographic absence of involvement of this region or can cause cortical bone perforation or erosion.^11^^,^ 19 In view of the biological behavior of the tumor, excision/enucleation is more prudent, followed by a complementary technique such as peripheral ostectomy or curettage. Large and invasive tumors may require more aggressive treatment, such as marginal or segmental resection.^13^ In the present case, although the tumor extended into the maxillary sinus and caused bulging of the buccal cortical bone extending between right upper premolars and first molar, a more conservative surgical approach was chosen considering not only effective treatment but also minimizing bone defects caused by the removal of the tumor.

Although CEOT is considered a benign tumor, rare cases of metastasis have been reported, especially to the cervical lymph nodes and lungs.^21^^,^ 22 Recurrences occur in approximately 14% of cases, and the prognosis of CEOT is considered good.^23^ In view of this possibility, the patient remains under follow-up and shows no signs of recurrence.

## CONCLUSIONS

The present patient had a CEOT at an uncommon anatomical site associated with a supernumerary tooth, representing a rare case given the findings described in the literature. The clinical-radiographic features of the tumor were similar to other odontogenic lesions. Thus, histopathological analysis is essential for establishing of the final diagnosis, which together with the clinical and imaging findings, will indicate the most appropriate therapeutic protocol for these tumors. Since CEOT associated with supernumerary teeth is rare, this case report adds to the literature and assists in establishing the epidemiological profile, biological behavior, and better therapeutic approaches for this tumor.
